# Evaluation of the Pharmaceutical Equivalence of Lidocaine and Prilocaine Creams

**DOI:** 10.3390/pharmaceutics18060707

**Published:** 2026-06-08

**Authors:** Peng Shao, Qiyu Feng, Fangfang Pan, Juan Zhang, Yalan Guan, Xiaoxia Sheng, Jinqi Zheng

**Affiliations:** 1 Zhejiang Key Laboratory of Biopharmaceutical Contact Materials, Zhejiang Institute for Food and Drug Control, 325, Pingle Street, Binjiang District, Hangzhou 310052, China; shaopeng@zjyj.org.cn (P.S.); panfangfang@zjyj.org.cn (F.P.); 2 Hangzhou Solipharma Co., Ltd., Building 1-F1, 260 6th Ave, Hangzhou 310018, China; fengqy@solipharma.com (Q.F.); zhangj@solipharma.com (J.Z.); guanyl@solipharma.com (Y.G.); shengxx@solipharma.com (X.S.); 3 Zhejiang Center for Drug & Cosmetic Evaluation, No. 140, Wensan Road, West Lake District, Hangzhou 310012, China

**Keywords:** lidocaine and prilocaine cream, topical formulation, in vitro–in vivo correlation, release rate, transdermal permeation

## Abstract

**Background:** Lidocaine and prilocaine cream is a compounded topical anesthetic formulation comprising lidocaine and prilocaine. Upon application, the active ingredients are locally released and permeate into the subcutaneous tissue, exerting anesthetic effects by blocking ion channels involved in nerve impulse transmission. Variations in drug permeation may influence the onset time, anesthetic efficacy, and duration of action. **Methods:** This study investigates the in vitro properties of the innovator formulation Emla^®^ and five generic formulations through in vitro bioequivalence studies and Q3 Characterization tests. **Results:** With the exception of TS, the generic formulations exhibited notable differences compared to the innovator product. Specifically, TB and TL demonstrated significantly higher in vitro release than Emla^®^, yet exhibited lower in vitro permeation rates. In contrast, TH and TT showed release rates comparable to Emla^®^, while their permeation rates were similarly reduced. **Conclusions:** These findings indicate that in vitro release rate does not directly predict in vitro permeation. Permeation behavior is significantly influenced by emulsion globule size, rheological characteristics (viscosity and elasticity), and pH, collectively underscoring the multifactorial nature of this phenomenon.

## 1. Introduction

The approval of topical generic drugs by the US FDA, EMA, and China’s NMPA has been primarily based on consistency of active ingredient content and the similarity of in vitro dissolution/release behavior, with certain categories supplemented by in vivo pharmacokinetic (PK) studies. However, this evaluation framework overlooks critical characteristics of topical formulations. Firstly, the efficacy of topical formulations depends on the local concentration-time profile of the active ingredient at the target skin site rather than systematic plasma drug concentration. Consequently, some generics that achieve plasma drug concentrations equivalent to the reference-listed drug (RLD) may fail to demonstrate therapeutic equivalence due to insufficient dermal retention resulting from differences in formulation matrices. Secondly, patient-centric attributes such as skin irritation, adhesiveness, spreadability, and residual sensation are not incorporated into current bioequivalence evaluation frameworks.

Although generic topical formulations meet the equivalence requirements for core efficacy indicators to the RLD, they may exhibit differences in clinical efficacy and patient experience due to variations in dosage form characteristics, manufacturing processes, and excipients [1,2]. To elucidate the origins of these subtle differences, it is essential to systematically compare Q3 characteristics between the generic products and the RLD, and to analyze how these differences affect the release and permeation of active ingredients during topical administration. Generally, topical formulations require continuous use to exert therapeutic effects, making subtle differences between generics and RLDs difficult to detect. As a topical formulation for local skin anesthesia, lidocaine–prilocaine exhibits rapid onset of action, enabling expedient clinical comparison between products. Therefore, we selected lidocaine–prilocaine topical formulation as the model system to systematically compare Q3 characteristics between the generic products and the RLD, providing a reference framework for generic development of topical formulations.

Lidocaine–prilocaine cream (25 mg lidocaine and 25 mg prilocaine per gram) was first approved by Swedish Medical Products Agency in 1984 under the trade name Emla^®^. Both active compounds are amide-type local anesthetics that stabilize neuronal membranes by inhibiting the ionic fluxes required for initiation and conduction of nerve impulses, thereby producing local anesthesia [3,4]. Anesthetic quality is determined by the duration of continuous application and the total dose delivered. This formulation is indicated for providing anesthesia prior to needle insertion and for superficial surgical procedures on skin and mucous membranes. Owing to the limited aqueous solubility of lidocaine and prilocaine [5], the actives are co-dissolved in polyoxyethylene (40) hydrogenated castor oil to form a eutectic mixture that enhances solubility. This mixture is subsequently dispersed into the swollen carbomer solution, and the pH is then adjusted with sodium hydroxide to yield the final cream. Carbomer provides the requisite viscosity and stabilizes the emulsified system. The pH influences the viscosity and rheological behavior of the carbomer dispersion, the extent of emulsification, and the resistance to migration of the active compounds, thereby governing the release rate of lidocaine and prilocaine from the formulation [6,7]. Emla^®^ is formulated to be weakly alkaline, neutralizing the skin’s weakly acidic barrier and facilitating active permeation.

The FDA issued the product-specific guidance for Lidocaine and Prilocaine Cream in 2014, followed by EMA releasing the “Guideline on Bioequivalence for Topical Preparations” in 2018. China subsequently published the “Technical Guideline for Bioequivalence Studies of Lidocaine and Prilocaine Cream” in 2024, specifying that Cmax (maximum plasma concentration), AUC0-t (area under the plasma concentration-time curve from time zero to the last measurable concentration), and AUC0-∞ (area under the plasma concentration-time curve from time zero to infinity) of lidocaine and prilocaine serve as the evaluation indicators for bioequivalence(BE) studies. Since then, numerous companies have introduced generic equivalents of Emla^®^ to the markets. In China, nearly 30 generic formulations are currently available. Clinical observations indicate variability in onset and duration of anesthesia among these products. While BE studies serve as the core basis for marketing approval of generic drugs, their design has inherent limitations that cannot fully capture clinical scenarios. BE studies primarily determine equivalence by comparing plasma concentration-time curves of the test and reference formulations in healthy volunteers. Lidocaine/prilocaine, however, is a topical medication whose efficacy directly depends on its concentration in local skin tissue, particularly the dermis, rather than systematic plasma concentration. In clinical practice, different products may exhibit identical plasma concentration profiles while differing in their distribution and accumulation within various skin layers, ultimately leading to variations in the onset time and clinical efficacy.

The present study conducted a series of comparative investigations to systematically evaluate the similarities and differences in drug release profiles and topical penetration kinetics between Emla^®^ and several generic formulations approved post 2024 acquired from China market. The objectives extend beyond merely identifying variations to elucidating their underlying causes from the pharmaceutical formulation and manufacturing processes perspectives.

## 2. Materials and Methods

### 2.1. Formulations

Formulations were obtained from the following suppliers: Aspen Pharma Trading Limited (Emla^®^, lot no. 100115; designated R; Karlskoga, Sweden), Manufacturer B (lot no. 026230920; TB), Manufacturer H (lot no. 230702; TH), Manufacturer L (lot no. 025231106; TL), Manufacturer S (lot no. 4250203; TS), and Manufacturer T (lot no. 230401; TT).

### 2.2. Reagents and Materials

Sodium chloride (analytical grade, Guangdong GHtech, Shantou, China) and sodium hydroxide (analytical grade, Guangdong GHtech, Shantou, China), gentamicin sulfate (analytical grade, Macklin, Shanghai, China) andmethanol (HPLC grade, BruderSCI, Shanghai, China) were used as obtained with further purification. Reference standards were lidocaine hydrochloride (93.1%, China National Institute for Food and Drug Control, Beijing, China) and prilocaine (98.5%, SINCO, Shenzhen, China). Bama miniature-pig skin was provided by Beijing Hebang Xingye Scientific Instrument Co., Ltd. (Beijing, China). The artificial PVDF membrane was purchased from Tianjin Jinteng Experiment Equipment Co., Ltd. (Tianjin, China). All solutions were prepared using deionized water (18 MΩ) purified with Milli-Q^®^ Reference system.

### 2.3. In Vitro Release Test Method

In vitro release was evaluated with a LOGAN SYSTEM 918-12 automated transdermal tester (Logan Instruments Corp., Somerset, NJ, USA). An artificial PVDF membrane (Tianjin Jinteng, Tianjin, China; 0.45 µm pore size) and a 6 mm-thick gasket were mounted in the diffusion cell, with 12 mL of isotonic saline served as receptor medium. Temperature was maintained at 32 °C and paddle speed at 600 rpm.

Approximately 1.0 g of each test cream (generic formulations, T; Emla^®^, R) was evenly applied onto an artificial membrane prior to assembly and fixed onto the diffusion cells. Six samples each of the generic and reference preparations were arranged in alternating order (“TRTRTR…” or “RTRTRT…”). The receptor compartment was charged with 12 mL of degassed isotonic saline and sealed to prevent evaporation or compositional change. At 0.5, 1, 2, 4 and 6 h, the entire receptor volume was withdrawn for analysis and immediately replaced with an equal volume of pre-equilibrated (32 °C) fresh medium.

### 2.4. Method for In Vitro Permeation Testing

In vitro permeation of the five generic formulations and Emla^®^ was assessed through excised skin from 1-month-old Bama miniature pigs. A LOGAN DSC-800 automated diffusion system (Logan Instruments Corp., Somerset, NJ, USA) was employed (32 °C, 600 rpm) with isotonic saline as receptor medium; 25 mg of cream was applied per cell. At predetermined time intervals, 1.5 mL samples were withdrawn for analysis. The volume of the receiving medium was 12 mL. After sampling, the entire medium was drained and immediately replaced with an equal volume of fresh medium pre-equilibrated at 32 °C.

### 2.5. Sample Analytical Method in Release and Permeation Testing

Reference standard solution: Accurately weighed lidocaine and prilocaine reference standards were dissolved in methanol and diluted to yield a mixed stock solution (approximately 1.25 mg mL^−1^ of each analyte). A 1 mL aliquot of the stock solution was transferred to a 10 mL volumetric flask, brought to volume with receptor fluid, and mixed to afford the working reference solution.

HPLC analysis was performed on a Thermo Scientific Ultimate 3000 (Dionex Softron GmbH, Germering, Germany) or Agilent 1260 Infinity II system (Agilent Technologies, Waldbronn, Germany) equipped with a C18 column (4.6 mm × 150 mm, 5 µm, COSMOSIL or equivalent). The mobile phase consisted of 0.5% ammonium dihydrogen phosphate (pH adjusted to 7.0 with 5 M NaOH)–methanol (40:60, *v*/*v*), delivered at 1.2 mL min^−1^. Column temperature was maintained at 35 °C and detection was carried out at 232 nm; injection volume was 10 µL.

### 2.6. Evaluation Method of In Vitro Release Test

Cumulative release per unit area was calculated from the standard curve using the following formulas [8]:

$$C_{n}=\frac{A_{n}-b}{k}$$

$$Q_{n}=\frac{C_{n}\times V+(C_{n-1}+C_{n-2}+\cdots +C_{1})\times V_{1}}{S}$$

where

C_n_ is the drug concentration in the receptor solution at the n-th sampling point of the sample solution, μg/mL;
b is the intercept of the obtained standard curve;
k is the slope of the obtained standard curve;
A_n_ is the peak area of the active ingredient measured at the n-th sampling point of the sample solution;
Q_n_ is the cumulative release amount per unit area at the n-th sampling point of the sample solution, in μg/cm^2^;
V is the volume of the receptor solution, 12 mL;
V_1_ is the sampling volume, 12 mL;
S is the application area, calculated as S = πr^2^ = 3.1415 × 0.75^2^ = 1.767 cm^2^.

Linear regression of cumulative release per unit area (Q) versus the square root of time (t½) was performed for each cell; the slope of the resulting line was taken as the in vitro release rate. Thirty-six individual release-rate ratios (T/R, where T = generic product and R = Emla^®^) were ranked in ascending order. The 8th and 29th ordered ratios define the non-parametric 90% confidence interval for the median T/R ratio. For bioequivalence acceptance, this 90% CI, expressed as a percentage, must lie entirely within 75~133.33%.

Result determination was conducted in accordance with the FDA guidelines [8]: Six samples each of the test and reference products were evaluated. For both the test and reference products, the relative standard deviation (RSD) of the release rate should not exceed 15% (*n* = 6), and the coefficient of determination (R^2^) should not be less than 0.97. The 90% confidence interval (CI) of the test and reference products (calculated by sorting 36 slope ratios in ascending order, where the 8th and 29th values represent the lower and upper limits of the 90% CI for the ratio of the median in vitro release rate of the test products to that of the reference products, respectively) should be within 75~133.33%.

If the above limits are exceeded, test an additional 12 samples each for the test and reference products. A total of 18 slopes per test products should be used to calculate the 90% CI (calculated by sorting 324 slope ratios in ascending order; the 110th and 215th ratios are the lower and upper limits of the 90% CI for the ratio of the median in vitro release rate of the test products to that of the reference products, respectively), which should be within 75~133.33%.

### 2.7. Evaluation Method of In Vitro Permeation Test

Permeation flux per unit area and cumulative amount permeated per unit area were derived from the standard curve using the following equations [8]:

$$J_{n}=\frac{V}{S\times (T_{n}-T_{n-1})}C_{n}$$

$$Q_{n}=\frac{V}{S}\sum_{n=1}^{n}C_{n}$$

where

Q_n_ is the cumulative amount permeated per unit area at the *n*-th sampling point (μg/cm^2^);
V is the volume of the receiving solution (5 mL);
S is the application area, S = πr^2^ = 3.1415 × 0.75^2^ = 1.767 cm^2^;
J_n_ is the permeation rate per unit area at the *n*-th sampling point (μg/cm^2^/h);
T_n_ is the time at the *n*-th sampling point (h);
T_n−1_ is the time at the (*n* − 1)-th sampling point (h).

The maximum permeation flux per unit area (J_max_) for each cell was identified from the individual J values; the cumulative amount permeated per unit area at the final sampling time was recorded as A_total_.

Generic (T) and originator (R) products were compared pair-wise, and T/R ratios were calculated for both J_max_ and A_total_. Non-parametric 90% confidence intervals for the median ratio were derived from the ranked data; limits were required to fall within 80.00~125.00%. In addition, the 90% CI of the geometric mean T/R ratio for both J_max_ and A_total_ had to lie within 80.00~125.00%.

The within-donor standard deviation for the originator product (S_WR_) was calculated based on the natural logarithm-transformed values of J_max_ and A_total_ [9]:

$${\left(\frac{\sum_{j=1}^{n}\sum_{i=1}^{r_{j}^{R}}{\left(R_{ij}-{\bar{R}}_{.j}\right)}^{2}}{r^{*}-n}\right)}^{1/2}$$

where

${\bar{R}}_{.j}=\frac{1}{r_{j}^{R}}\sum_{i=1}^{r_{j}^{R}}R_{ij}$ is the mean of the natural log-transformed results for all $r_{j}^{R}$ replicates from the j-th donor of the originator product;
$R_{ij}$ is the natural log-transformed IVPT endpoint (J_max_ or A_total_) for the i-th skin section from the j-th donor of the originator product;
$r^{*}=r_{1}^{R}+\cdots +r_{n}^{R}$ is the total number of skin sections in the R group;
n: Number of donors.

Average bioequivalence (ABE) was assessed: the 90% confidence interval for the difference in means of log-transformed J_max_ and A_total_ (*μ*_*T*_ − *μ*_*R*_) was required to lie within 80.00–125.00%.

### 2.8. Method for Emulsion Globule Size Distribution

Emulsion globule size distribution was determined by optical microscopy (XPV-990, Shanghai Changfang Optical instrument Co., Ltd., Shanghai, China). A small aliquot of cream was placed on a glass slide, covered with a coverslip, and gently pressed to obtain a thin layer. Droplet diameters (D_10_, D_50_, D_90_) were quantified at 400× magnification using image analysis software (ZML310 Transdermal Cream Particle Analysis Software/equivalent, Zimeng Technology, Shanghai, China).

### 2.9. Method for Rheological Behavior

The elastic modulus was determined using a HAAKE MARS 60 rotational rheometer (Thermo Electron GmbH, Karlsruhe, Germany). An appropriate aliquot of sample (approximately 0.7 mL for the P25/Ti rotor) was loaded at the center of the plate. The rotor was subsequently lowered to its minimum position to ensure complete filling of the cone-plate gap. Excess material was carefully trimmed using a spatula. Oscillatory frequency sweep measurements were conducted over an angular frequency range of 1–100 rad/s at 25 °C with a constant stress of 5 Pa.

### 2.10. Method for Viscosity Test

The viscosity measurements were performed using a Brookfield DV2TRV rotational viscometer (Brookfield Engineering Laboratories, Inc., Middleboro, MA, USA). A sample aliquot of approximately 13.5 g was loaded into the sample chamber. The instrument parameters were configured as follows: SC4-29 spindle, rotational speed of 3 rpm, test temperature maintained at 25 °C ± 1 °C, with a test duration of 3 min and data acquisition at 1 min intervals using multi-point averaging mode.

### 2.11. Method for pH Test

pH was measured at 25 °C using a Mettler Toledo FE28 (Mettler Toledo, Shanghai, China) or SevenDirect SD20 pH meter (Mettler Toledo, Shanghai, China). Two independent readings were taken (the difference between the pH readings should not exceed 0.1) and averaged to give the reported value.

## 3. Results and Discussion

### 3.1. In Vitro Release Testing (IVRT)

**Figure 1:**
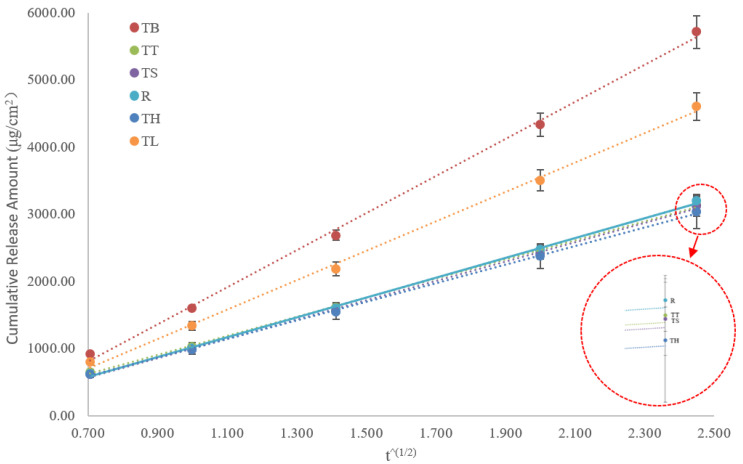
Mean release profile of lidocaine.

**Figure 2:**
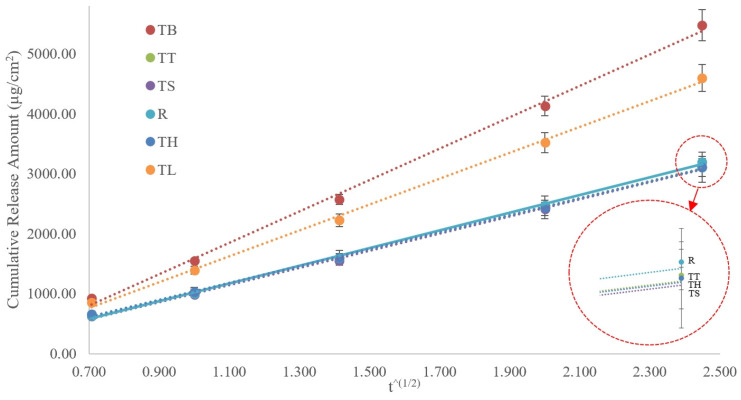
Mean release profile of prilocaine.

The IVRT results for the generic formulations and the reference product Emla^®^ are presented in Table 1. Comparison analysis of the mean release rates revealed significant differences between some of the generic formulations and the reference product, especially TB and TL. For lidocaine, the K_T_/K_R_ values (the ratio of the mean release rate of the generic test to the reference) across the five generic formulations ranged from 0.94 to 1.86 (mean release profile shown in Figure 1). Similarly, for prilocaine, the K_T_/K_R_ values ranged from 0.96 to 1.78 (mean release profile shown in Figure 2).

Notably, lidocaine and prilocaine exhibited highly congruent release kinetics, with the ratio of their release rates (KL/KP, where KL represents the release rate of lidocaine and KP that of prilocaine) ranging from 0.98 to 1.05. This observed similarity in release behavior is likely attributable to their comparable solubility profiles, as indicated by their respective LogP values (lidocaine = 2.44; prilocaine = 2.1), and their identical physical state within the formulation, with both active ingredients dissolved in the primary vehicle, polyoxyethylene (40) hydrogenated castor oil [10,11].

### 3.2. In Vitro Permeation Testing (IVPT)

The IVPT results for the generic formulations and the reference product Emla^®^ are summarized in Table 2. Comparison analysis of the two active ingredients reveals noticeable differences between the generic formulations and the reference product in terms of both the cumulative amount permeated per unit area and the maximum permeation rate per unit area.

**Figure 3:**
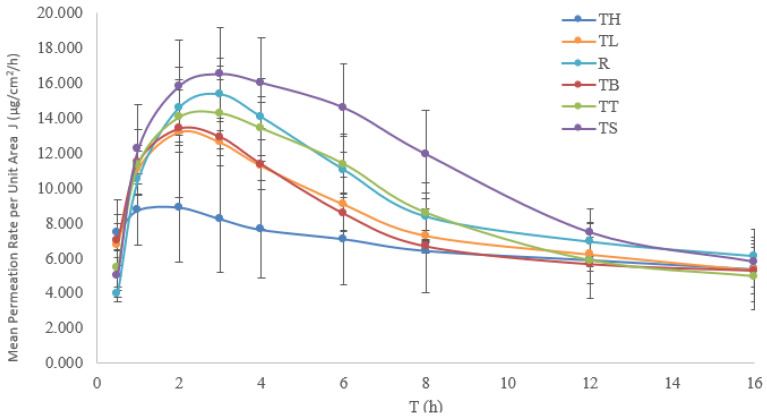
Mean permeation rate per unit area curve of lidocaine.

**Figure 4:**
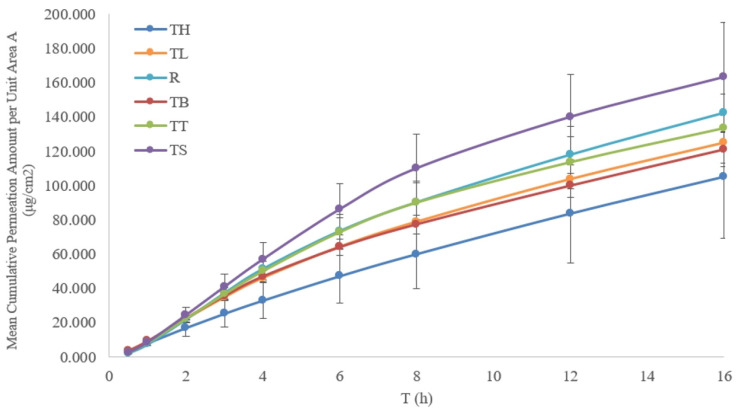
Mean cumulative amount permeated per unit area curve of lidocaine.

For lidocaine, the A_T_/A_R_ values (the ratio of the cumulative amount permeated per unit area of generic test to the reference) among the five generic formulations ranged from 0.74 to 1.15, while the J_T_/J_R_ values (the ratio of the maximum permeation rate per unit area of the generic test to the reference) ranged from 0.61 to 1.08. The corresponding average permeation rate and cumulative permeation amount profiles per unit area for lidocaine are illustrated in Figure 3 and Figure 4, respectively.

**Figure 5:**
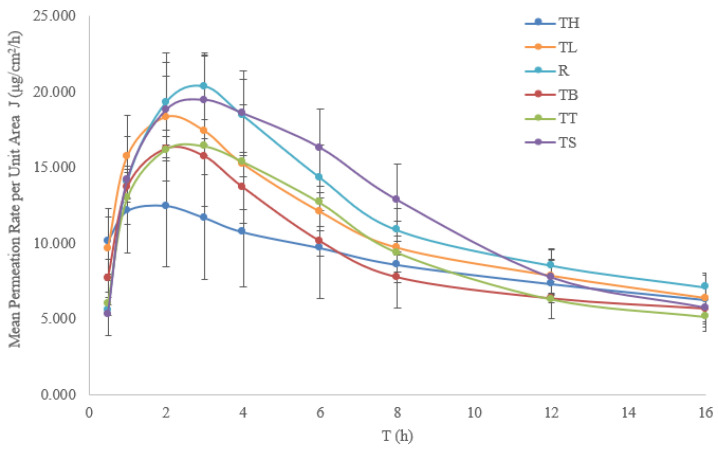
Mean permeation rate per unit area curve of prilocaine.

**Figure 6:**
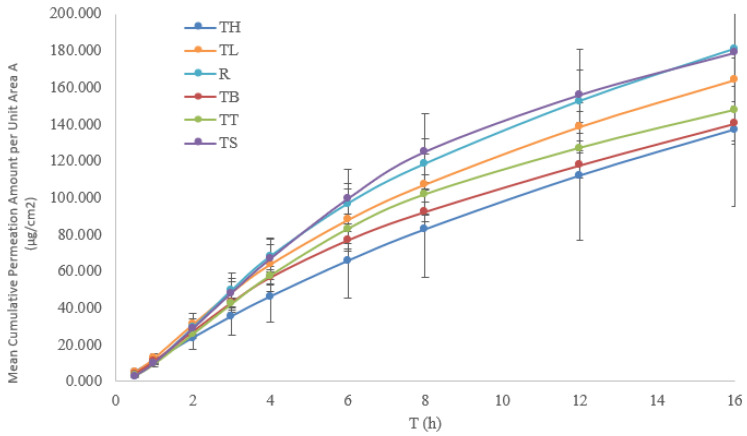
Mean cumulative amount permeated per unit area curve of prilocaine.

For prilocaine, the A_T_/A_R_ values ranged from 0.76 to 0.99, and the J_T_/J_R_ values ranged from 0.64 to 0.96. The average permeation rate and cumulative permeation amount profiles per unit area for prilocaine are shown in Figure 5 and Figure 6, respectively.

Additionally, the transdermal permeation extent of lidocaine was significantly lower than that of prilocaine. The ratio of cumulative permeation amount per unit area between lidocaine and prilocaine (A_L_/A_P_) ranged from 0.76 to 0.91, while the ratio of their maximum permeation rates per unit area (J_L_/J_P_) ranged from 0.72 to 0.87. These results collectively indicate that prilocaine has better transdermal permeability than lidocaine. This difference may be attributed to lidocaine’s stronger protein binding affinity, which consequently limits its transdermal permeation ability [12,13].

### 3.3. Differences Between Generic Formulations and Emla^®^

To evaluate the comparative performance of generic formulations against the originator, the release rate (K), cumulative amount permeated per unit area (A), and maximum permeation rate per unit area (J) for each generic product were calculated as ratios (T/R) relative to the corresponding parameters of the reference formulation. The results are summarized in Table 3, revealing formulation-dependent variability in consistency with the originator product, as detailed below:

Formulation TS demonstrated comparable performance to the originator Emla^®^, with all three parameter ratios (T/R) falling within the acceptable range of 80.00~125.00% and confidence intervals (CIs) for both IVRT and IVPT tests meeting the predefined criteria for consistency evaluation.

In contrast, formulations TL and TB exhibited significantly higher release rates than the reference product, with IVRT CIs exceeding the acceptance range of 75~133.33%. However, both cumulative permeation (A) and maximum flux (J) for these formulations were lower than the originator, with IVPT CIs falling outside the range of 80.00~125.00%.

Formulation TT showed comparable release characteristics to the reference, with IVRT CIs meeting consistency requirements. However, both permeation parameters (A and J) were reduced relative to the originator, and while the IVPT CI for lidocaine met acceptance criteria, prilocaine fell outside 80.00~125.00%.

Similarly, formulation TH demonstrated equivalent release performance with acceptable IVRT CIs but showed reduced transdermal permeation parameters with IVPT CIs outside 80.00~125.00%.

### 3.4. Emulsion Globule Size

**Figure 7:**
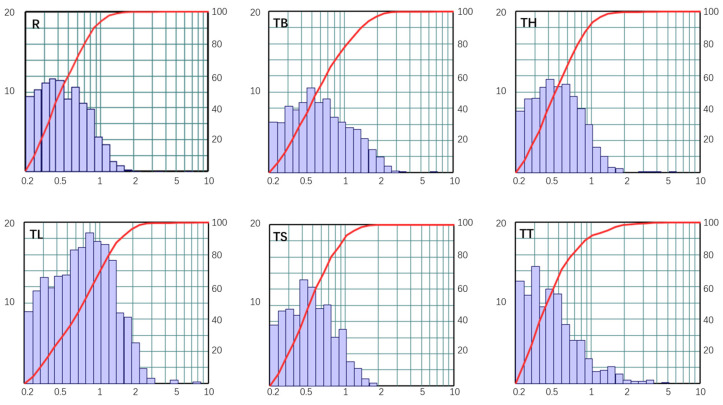
The globule size distribution of the generic formulations R, TB, TH, TL, TS, and TT.

The globule size distributions of the investigated formulations are presented in Figure 7 and Table 4. The D_10_, D_50_, and D_90_ values, as determined by particle size analysis software, are summarized in Table 4. Analysis of these data indicates that the globule sizes of generic formulations TH, TS, and TT were comparable to that of the originator formulation. In contrast, formulations TB and TL exhibited larger average globule sizes of 0.72 μm and 0.83 μm, respectively, exceeding that of the originator formulation (0.51 μm).

Increased average globule size may originate from reduced emulsification efficiency, variations in other critical process parameters, excipient quality attributes or pH of the formulation TL and TB which will be discussed in Section 3.5. Based on the results, TL and TB exhibit higher elastic modulus, consequently impairing homogenization effectiveness [14,15].

The observed higher release rates from TB and TL, despite their larger globule sizes, appear counterintuitive since smaller droplets typically exhibit faster release due to greater surface area-to-volume ratios. This anomalous behavior may be attributed to formulation instability or altered physicochemical properties. As globule size increases, the surface density of surfactant molecules decreases, potentially compromising the stability of the interfacial layer [16,17]. This reduced interfacial stability may lead to emulsion destabilization or altered drug partitioning behavior, contributing to the accelerated release observed in vitro. However, such enlarged droplets exhibit locally reduced spreading ability on the skin surface, thereby decreasing the contact area between the drug and the skin. This ultimately leads to reduced transdermal penetration and total permeation, despite the accelerated in vitro release rate. The discrepancy between in vitro release and in vivo permeation underscores the importance of skin interaction and spreading characteristics in determining transdermal delivery performance.

### 3.5. Other Q3 Characteristics

The Q3 equivalence requirements for topical formulations necessitate that generic drugs possess identical formulation composition and content as the reference-listed drug, along with the same microstructural and rheological properties, as well as matching in vitro release characteristics. Therefore, in addition to IVPT, IVRT, and droplet size, other Q3 characteristics between Emla^®^ and the generic formulations were investigated, including viscosity, elastic modulus and pH. The results are presented in Table 5.

#### 3.5.1. Effect of Viscosity

Viscosity directly influences the diffusion resistance encountered by active ingredient-containing oil droplets, whereby elevated viscosity may reduce both transdermal flux and total permeation amount. In high-viscosity systems, molecular chains form densely entangled networks that encapsulate drug molecules, thereby restricting their diffusion toward the formulation boundary. This increased viscosity diminishes system fluidity and lowers the diffusion coefficient of drug molecules, significantly reducing their mean displacement per unit time. Consequently, drug migration from the formulation interior to the drug/skin interface is retarded, potentially resulting in incomplete release of the active component. Furthermore, high-viscosity formulations demonstrate compromised spreadability, preventing uniform distribution across the microtopography of the skin surface. This leads to discontinuous drug-skin contact and a substantial decrease in effective contact area [18,19].

The viscosity characteristics of the investigated formulations are presented in Table 5. Correlation with the data in Table 3 demonstrates the considerable influence of viscosity on both drug release and transdermal permeation. Formulation TH exhibited viscosity comparable to the originator product. Formulations TB, TL, and TS showed moderately elevated viscosity relative to the reference, whereas formulation TT demonstrated approximately 1.9-fold higher viscosity than the originator. This substantial viscosity increase in formulation TT creates a diffusional barrier that limits drug availability at the skin surface. Additionally, the substantially lower pH of TT formulation, as will be explained in Section 3.5.3, also increases ionization of both active ingredients and impairs lipid membrane permeation. The combination of high ionization and increased viscosity results in comparable IVRT but reduced IVPT.

#### 3.5.2. Effect of Elastic Modulus

The elastic modulus governs the structural integrity of active ingredient-containing formulations. Topical systems with elevated elastic modulus demonstrate reduced transdermal permeation parameters, as high elasticity induces structural rigidity and limited deformability, thereby compromising essential conditions for drug release, skin contact, and permeation pathways. The microtextured skin surface undergoes continuous deformation during movement, requiring formulations to maintain intimate interfacial contact for effective drug delivery. However, highly elastic systems exhibit insufficient deformation capacity to adapt to cutaneous microtopography, resulting in substantially diminished effective contact area. Furthermore, elevated elastic modulus typically arises from extensive cross-linking within the polymer matrix, such as through high concentrations of carbomer or hydroxypropyl methylcellulose. The resulting dense network structure immobilizes drug molecules, significantly impeding their diffusion through the rigid polymeric architecture [20,21]. Consequently, an increase in elastic modulus can negatively impact both transdermal flux and total permeation.

The elastic modulus values for the test formulations are summarized in Table 5. Integration of these results with the data presented in Table 3 reveals a discernible influence of elastic modulus on both drug release and permeation characteristics. Generic formulations TS and TT exhibited elastic moduli comparable to the originator product, while formulation TH demonstrated a moderately reduced modulus.

In contrast, formulations TL and TB showed substantially elevated elastic moduli relative to the reference standard. This significant increase in elastic modulus creates several barriers to skin permeation:

- Reduced spreadability: stiffer formulations spread poorly on skin, thereby decreasing contact area.
- Impaired drug mobility: higher structural resistance limits drug diffusion within the formulation toward the skin surface.
- Poor skin–formulation interface: rigid formulations cannot conform to skin microstructure, reducing effective contact area.

Furthermore, both TL and TB formulations exhibit a lower pH than the RLD. As discussed in Section 3.5.3, a lower pH not only enhances drug release but also hinders permeation. The combined effects of high modulus and lower pH therefore contribute to the observed reduction in permeation.

#### 3.5.3. Effect of pH

The transdermal performance of lidocaine and prilocaine, characterized by maximum flux (J) and cumulative permeation amount (A), demonstrated no direct correlation with release rate (K) but exhibited strong pH dependence. Specifically, the reference product (Emla^®^) and generic formulation TS—both possessing higher pH values—achieved superior J and A values. Conversely, all other generic formulations with lower pH exhibited diminished transdermal performance. Notably, despite the substantially higher release rates of formulations TB and TL compared to the reference, their transdermal parameters remained comparatively low.

Under physiological conditions, the skin surface and stratum corneum maintain an acidic pH range of 4–6, which is essential for preserving stratum corneum integrity and epidermal barrier homeostasis [22,23]. The stratum corneum, organized in a “brick-and-mortar” structure of corneocytes and intercellular lipids, represents the primary barrier to transdermal permeation. Alkaline substances can compromise this barrier through structural disruption: upon penetration, compounds with pH > 9 alter intracellular acid-base balance. The normal weakly acidic intracellular environment (pH ≈ 7.2) becomes neutralized by alkaline components, causing osmotic imbalance. Subsequent osmotic pressure changes drive water diffusion from intracellular to extracellular compartments, inducing cellular dehydration and corneocyte shrinkage. This shrinkage loosens intercellular connections, creating gaps in the dense “brick-and-mortar” organization and significantly expanding intercellular spaces. These enlarged spaces establish new permeation pathways, enabling rapid drug diffusion toward the dermis without requiring penetration of the intact lipid barrier [24,25].

Furthermore, lidocaine (pKa ≈ 7.85) and prilocaine (pKa ≈ 7.7) are weak bases that exist predominantly in their unionized (free base) form at pH values above their respective pKa values. At the formulation pH (8.8–8.9 for TT and TH vs. 9.2 for R), both active ingredients are primarily unionized; however, the lower pH of TT and TH results in a greater proportion of the ionized species compared to R. During IVRT, this increased ionized fraction enhances aqueous solubility and promotes partitioning into an aqueous receptor medium during IVRT, thereby yielding higher release rates. Conversely, as the skin stratum corneum is lipophilic, drug in the molecular form is more conducive to skin penetration compared to the ionic form. Thus, while the lower pH of formulation TH and TT favors drug release, it simultaneously impairs lipid membrane permeation. To achieve effective transdermal delivery, the formulation pH must be elevated further to shift the equilibrium toward the unionized free base form, thereby facilitating efficient skin permeation across stratum corneum [26].

## 4. Conclusions

The drug release and permeation of semisolid formulations are governed by the complex interplay of compositional characteristics, microstructural properties (e.g., emulsion globule-size distribution), and rheological behavior. Developing bioequivalent generic products therefore require comprehensive characterization and integrated optimization of these critical attributes to ensure therapeutic equivalence with the reference-listed drug.

This study demonstrates that pharmacokinetic bioequivalence (PK-BE) does not guarantee identical in vitro release or transdermal permeation performance among generic lidocaine/prilocaine creams. The observed variations confirm that critical physicochemical attributes—pH, emulsion globule size, viscosity, and elastic modulus—exert synergistic influence on mass transfer kinetics. Crucially, in vitro release profiles alone are insufficient predictors of transdermal permeation efficacy, highlighting inherent limitations in current bioequivalence frameworks for complex topical formulations.

These findings carry significant implications for both clinical practice and generic drug development. Clinicians should remain vigilant regarding potential therapeutic variability among bioequivalent products to ensure precise medication use and patient safety. For developers, achieving true therapeutic equivalence requires strict adherence to Q1, Q2, and particularly Q3 sameness, rather than merely meeting PK-BE criteria. For topical formulations containing ionizable active ingredients, more stringent consistency standards must be established for pH and microstructure-related critical quality attributes. Furthermore, analytical methods with higher discriminatory power should be developed, and manufacturing process parameters must be systematically investigated as key determinants of formulation performance. Ultimately, ensuring batch-to-batch consistency and alignment with the reference-listed drug in both release behavior and transdermal permeation is essential for delivering high-quality generic topical products with reliable therapeutic equivalence.

## Figures and Tables

**Table 1:** Results of IVRT.

Ingredient	Specification	R	TB	TH	TL	TS	TT
Lidocaine	K	1478.47	2757.13	1388.02	2188.79	1439.49	1421.36
	K_T_/K_R_	/	1.86	0.94	1.48	0.97	0.96
	C_L_ (%)	/	176.03	85.55	142.79	95.41	92.67
	C_U_ (%)	/	194.21	100.59	156.88	99.51	102.22
Prilocaine	K	1473.83	2619.51	1410.69	2153.92	1429.24	1419.77
	K_T_/K_R_	/	1.78	0.96	1.46	0.97	0.96
	C_L_ (%)	/	167.67	87.62	140.65	95.44	92.79
	C_U_ (%)	/	185.09	103.22	155.93	99.70	103.08
K_lidocaine_/K_prilocaine_		1.00	1.05	0.98	1.02	1.01	1.00

Note: K—mean release rate (μg/cm^2^/h^1/2^); C_L_—lower confidence limit; C_U_—upper confidence limit; K_T_/K_R_—ratio of the mean release rate between the generic and originator formulations; K_lidocaine_/K_prilocaine_—ratio of the release rates of lidocaine and prilocaine.

**Table 2:** Results of IVPT.

Ingredient	Specification	R	TB	TH	TL	TS	TT
Lidocaine	A	142.121	120.953	104.982	124.825	163.044	133.213
	A_T_/A_R_	/	0.85	0.74	0.88	1.15	0.94
	C_LA_ (%)	/	80.93	59.89	81.29	106.40	90.20
	C_UA_ (%)	/	90.91	91.68	96.85	122.71	97.61
	J	15.343	13.43	9.335	13.262	16.491	14.378
	J_T_/J_R_	/	0.88	0.61	0.86	1.08	0.94
	C_LJ_ (%)	/	82.97	50.99	74.46	103.45	90.35
	C_UJ_ (%)	/	94.11	73.64	99.15	111.20	97.69
Prilocaine	A	180.737	140.226	136.977	164.027	178.804	147.467
	A_T_/A_R_	/	0.78	0.76	0.91	0.99	0.82
	C_LA_ (%)	/	74.54	63.03	84.98	93.15	78.53
	C_UA_ (%)	/	81.55	91.02	98.48	104.13	84.89
	J	20.350	16.306	12.980	18.358	19.496	16.564
	J_T_/J_R_	/	0.80	0.64	0.90	0.96	0.81
	C_LJ_ (%)	/	76.70	54.38	79.93	92.70	79.27
	C_UJ_ (%)	/	84.73	75.29	101.10	98.26	83.73
A_lidocaine_/A_prilocaine_		0.79	0.86	0.77	0.76	0.91	0.90
J_lidocaine_/J_prilocaine_		0.75	0.82	0.72	0.72	0.85	0.87

Note: A—A_toal_, cumulative amount permeated per unit area (μg/cm^2^); J—J_max_, maximum permeation rate per unit area (μg/cm^2^/h); C_L*_—lower confidence limit (* indicates A and J, same below); C_U*_—upper confidence limit; A_T_/A_R_—ratio of the cumulative amount permeated per unit area between the generic and originator formulations; J_T_/J_R_—ratio of the maximum permeation rate between the generic and originator formulations; A_lidocaine_/A_prilocaine_—ratio of the cumulative amount permeated per unit area between lidocaine and prilocaine; J_lidocaine_/J_prilocaine_—ratio of the maximum permeation rates per unit area between lidocaine and prilocaine.

**Table 3:** Comparison of K, A, J ratios between generic formulations and Emla^®^.

	Formulation	R	TB	TH	TL	TS	TT
Specification							
K_lidocaine_		1	1.86	0.94	1.48	0.97	0.96
K_prilocaine_		1	1.78	0.96	1.46	0.97	0.96
J_lidocaine_		1	0.88	0.61	0.86	1.08	0.94
J_prilocaine_		1	0.8	0.64	0.9	0.96	0.81
A_lidocaine_		1	0.85	0.74	0.88	1.15	0.94
A_prilocaine_		1	0.78	0.76	0.91	0.99	0.82

**Table 4:** Test results of emulsion globule size.

Specification	R	TB	TH	TL	TS	TT
D_10_	0.24	0.26	0.25	0.28	0.25	0.23
D_50_	0.43	0.56	0.46	0.70	0.47	0.39
D_90_	0.87	1.43	0.92	1.55	1.01	0.93
Average	0.51	0.72	0.55	0.83	0.57	0.53

**Table 5:** Test results of viscosity, elastic modulus, and pH.

Specification	R	TB	TH	TL	TS	TT
pH	9.2	8.8	8.9	8.9	9.1	8.4
Viscosity (Pa·s)	108	150	115	163	156	195
1 rad/s Elastic Modulus G′ (Pa)	465.16	574	406.5	624	477.9	444.4
100 rad/s Elastic Modulus G′ (Pa)	641.53	763.2	575.9	822.1	725.2	683.9

## Data Availability

The original contributions presented in this study are included in the article. Further inquiries can be directed to the corresponding authors.

## Abbreviations

The following abbreviations are used in this manuscript:

PK	pharmacokinetic
RLD	reference-listed drug
FDA	U.S. Food and Drug Administration
EMA	European Medicines Agency
NMPA	National Medical Products Administration
C_max_	maximum plasma concentration
AUC_0-t_	area under the plasma concentration-time curve from time zero to the last measurable concentration)
AUC_0-∞_	area under the plasma concentration-time curve from time zero to infinity
HPLC	High Performance Liquid Chromatography
ABE	Average bioequivalence
IVRT	In Vitro Release Testing
IVPT	In Vitro Permeation Testing
K	release rate
A	cumulative amount permeated per unit area
J	maximum permeation rate per unit area
CI	confidence interval
